# Murine Models of Myelofibrosis

**DOI:** 10.3390/cancers12092381

**Published:** 2020-08-23

**Authors:** Sebastien Jacquelin, Frederike Kramer, Ann Mullally, Steven W. Lane

**Affiliations:** 1 Cancer program QIMR Berghofer Medical Research Institute, Brisbane, Queensland 4006, Australia; 2 Division of Hematology, Department of Medicine, Brigham and Women’s Hospital, Harvard Medical School, Boston, MA 02115, USA; fkramer2@bwh.harvard.edu (F.K.); ann_mullally@dfci.harvard.edu (A.M.); 3 Cancer Care Services, The Royal Brisbane and Women’s Hospital, Brisbane 4029, Australia; 4 University of Queensland, St Lucia, QLD 4072, Australia

**Keywords:** myeloproliferative neoplasm, MPN, mouse model, myelofibrosis, cancer, hematopoietic stem cells, oncogene, mutation

## Abstract

Myelofibrosis (MF) is subtype of myeloproliferative neoplasm (MPN) characterized by a relatively poor prognosis in patients. Understanding the factors that drive MF pathogenesis is crucial to identifying novel therapeutic approaches with the potential to improve patient care. Driver mutations in three main genes (janus kinase 2 (*JAK2*), calreticulin (*CALR*), and myeloproliferative leukemia virus oncogene (*MPL*)) are recurrently mutated in MPN and are sufficient to engender MPN using animal models. Interestingly, animal studies have shown that the underlying molecular mutation and the acquisition of additional genetic lesions is associated with MF outcome and transition from early stage MPN such as essential thrombocythemia (ET) and polycythemia vera (PV) to secondary MF. In this issue, we review murine models that have contributed to a better characterization of MF pathobiology and identification of new therapeutic opportunities in MPN.

## 1. Introduction

Myeloproliferative neoplasms (MPN) are a group of myeloid-derived malignancies that lead to excessive production of blood cells (erythrocytes, leukocytes, or platelets). Genetic gain of function mutations drive abnormal clonal proliferation of myeloid progenitors, leading to accumulation of fully differentiated hematopoietic elements. Unlike in acute myeloid leukemia (AML), MPN hematopoietic progenitors retain a relatively normal differentiation program. However, they are characterized by hypersensitivity to cytokines, in particular, to erythropoietin (EPO) and thrombopoietin (TPO) [1,2]. Three MPN subtypes have been identified: essential thrombocythemia (ET), polycythemia vera (PV), and primary myelofibrosis (PMF). PMF was previously known as chronic idiopathic myelofibrosis (CIMF), agnogenic myeloid metaplasia (AMM), and myelofibrosis with myeloid metaplasia (MMM). In 2007, the International Working Group for Myelofibrosis Research and Treatment (IWG-MRT) standardized the nomenclature referring to PMF [3]. Myelofibrosis arising secondary to PV or ET have since been named post-PV MF and post-ET MF, respectively. PMF is the least frequent of the three classic MPN but is the most aggressive and is associated with a significantly shortened survival [4,5]. The abbreviation MF will be used in this review to denote PMF and post ET/PV MF. MF is defined as proliferation, atypia of megakaryocytes (MK), and bone marrow (reticulin and/or collagen) fibrosis (major diagnostic criteria) with additional features of extramedullary hematopoiesis, splenomegaly, and abnormal cytokine expression.

The vast majority of patients carry mutations that activate the JAK-STAT signaling pathway, namely janus kinase 2 (*JAK2*), calreticulin (*CALR*), and myeloproliferative leukemia virus oncogene (*MPL)*. In PMF, *JAK2*^V617F^ mutations are the most common (58%), followed by *CALR* (25%) and *MPL* (7%) [5,6,7]. Constitutive or aberrant activation of JAK-STAT signaling plays a pivotal role in the pathogenesis of MF. Following ligand receptor binding to cytokine receptors such as EPOR or MPL, homodimeric JAK2 signaling molecules activate downstream signaling pathways, leading to the phosphorylation of transcription factor Stat5. This in turn triggers nuclear translocation of these proteins and activation of multiple targets including inflammatory and survival-related genes [8,9,10].

**Figure 1:**
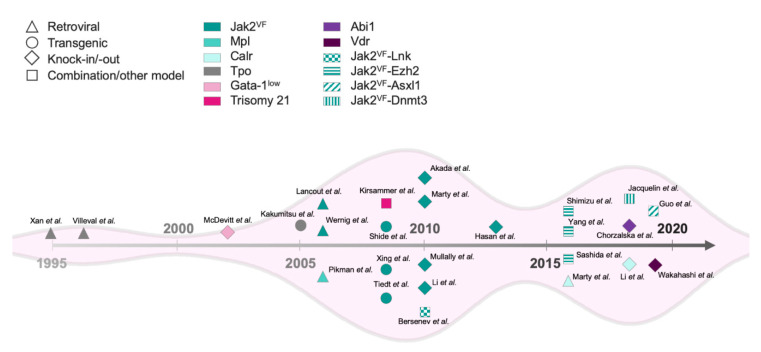
Timeline of published myelofibrosis mouse models.

Aberrant inflammatory cytokines and signaling molecules affect both hematopoietic stem cells (HSC) and stromal cells within the hematopoietic niche. Little is known about the cellular targets of bone marrow fibrosis; however, mesenchymal stromal cells (MSC) from pre-fibrotic MPN patients can increase extracellular matrix remodeling, can decrease hematopoiesis supporting capacity, and can thus play a role in MF pathology [11]. In this review, we will review murine models of MF (Figure 1), discuss new findings that shed light on the underlying molecular mechanisms, and identify therapeutic opportunities that might improve outcomes in this challenging disease.

## 2. JAK2^V617F^

JAK2 is a non-receptor tyrosine kinase that mediates cytokine receptor signaling pathways downstream of MPL and the EPO receptor, where it homodimerizes to initiate STAT1,3,5-dependent signaling in response to EPO-EPOR or TPO-MPL binding. JAK2 also signals downstream of numerous other cytokines including interleukin (IL) 3, granulocyte-macrophage colony-stimulating factor (GM-CSF), and interferon γ (IFNγ) [12]. An activating mutation in JAK2 which involves a valine to phenylalanine substitution *JAK2^V617F^* was first identified in 2005 and is the most prevalent mutation in MPN, found in 95% of PV, and in more than 50% of PMF and ET [1,13,14,15,16]

Following the discovery of the *JAK2^V617^*^F^ mutation (*JAK2^VF^*), retroviral models were generated to assess the phenotypic effects of this mutation in vivo [1,17,18,19,20] (Table 1). Mice transplanted with *JAK2^VF^* expressing donor bone marrow developed a phenotype resembling PV, but MF transformation was described in two studies [18,19]. Common MPN features such as high hemoglobin, leukocytosis, and megakaryocyte hyperplasia were observed at early stages in these two studies and splenomegaly was identified at endpoints, consistent with early PV progressing to MF. However, Lacout and colleagues described increased fibrosis at later time points (>4 months) after transplantation that were associated with marrow hypocellularity, anemia, thrombocytopenia, and granulocytosis, more reminiscent of PMF features. Wernig et al. observed MF in Balb/c but not in C57/Bl6 recipients after retroviral expression of *JAK2^VF^*, suggesting that additional host factors may regulate the development of fibrosis [19].

MF transformation potentiation could be explained by different plasmid backbone (pMEGIX vs. MSCV-IRES-EGFP), transfection efficiencies, or possibly expression level of the *Jak2^VF^* transgene. Of note, in the study of Lacout et al., phenotypic MF was transplantable into secondary recipients while, in the model generated by Wernig et al., it was not [18,19].

Although informative, retroviral models engender marked overexpression of *JAK2V617F,* and to more closely recapitulate physiological level expression, genetically engineered mouse models (GEMM) of *JAK2^VF^* were developed (previously reviewed in detail [25,26]). The development of GEMM models allowed correlation of disease phenotype with different gene expression levels, resulting in the conclusion that *JAK2^VF^* expression levels influence the MPN phenotype. In one transgenic mouse model, Shide et al. were able to demonstrate that *Jak2^VF^* expression could lead to MF transformation when *Jak2^VF^* expression was higher than wild-type (WT) Jak2 expression [27]. In a second transgenic model, Xing et al. expressed *Jak2^VF^* under the control of the *vav* promoter [28], resulting in higher levels of *Jak2^VF^* expression and age-related MF in 38% of *Jak2^VF^* mice.

Five *Jak2^VF^* knock-in models were published [29,30,31,32] in which murine *Jak2^VF^* from the endogenous murine *Jak2* promoter was expressed in different manners (Table 2). Akada et al. and Mullally et al. generated conditional *Jak2^VF^* knock-in models [29,32] while *Jak2^VF^* expression was constitutive in the model generated by Marty et al. [31]. Hasan and colleagues generated conditional *Jak2^VF^* mice, where mutant Jak2 was expressed from the endogenous locus using the VavCre system [33]; the same model was later used by Mansier et al. using recombination by PF4Cre [34]. Li et al. developed a model within which human *JAK2^VF^* was conditionally expressed from the endogenous murine *Jak2* promoter. In all these models, erythrocytosis, leukocytosis, extramedullary hematopoiesis, and MF development (in a minority of mice after 26 weeks) were observed. Interestingly, in the conditional *Jak2^VF^* knock-in model we developed, we have not observed MF in primary mice (likely due to thrombosis-related decreased survival); however, late progression to MF is infrequently observed in secondary recipients [35]. Once again, heterogeneity in the fibrosis phenotype between the *Jak2^VF^* knock-in models may relate to the level of *Jak2^VF^* expression.

## 3. *MPL* Mutation (W515L and W515K)

In 2006, point mutations in the TPO receptor MPL were discovered among JAK2^VF^-negative MPN [21]. Somatic mutations in MPL^W515L/K^ have been found in 5% and 1% respectively of MF and ET patients [41]. Pikman et al. used MPL^W515L^ expressed retrovirally in hematopoietic stem and progenitor cells (HSPC) to induce MPN characterized by leukocytosis, thrombocytosis, splenomegaly, and reticulin fibrosis [21] (Table 1). MPL^W515L^ expression recapitulated phenotypic features of human MF such as megakaryocytic hyperplasia, splenomegaly, extra medullary hematopoiesis, and thrombocytosis. Using this model, Kleppe et al. showed that JAK-STAT activation (STAT3) and cytokine production is found in malignant and nonmalignant hematopoietic cells in MF [9]. MPL^W515L^ -mutant HSPC were the largest source of IL6; however, mutant and WT cells secreted tumor necrosis factor (TNF), IL10, and C-C motif chemokine 2 (CCL2). Global cytokine production and the proportion of cells secreting multiple cytokines were increased in differentiated cells (both mutant and nonmutant) and in MPL^W515L^ mutant-expressing HSPC from MF mice. These data suggest a potential cross talk between malignant and nonmalignant cells and show that cytokine production by both populations is an important feature of MF. The authors identified six cytokines (IL6, IL10, IL12, TNFα, CCL2, and CCL5) aberrantly produced in their murine models, which were also found to be increased in granulocytes from patients with MF (as compared to healthy individuals). This work provided preclinical evidence in support of the use of JAK2 inhibitors in MPN patients positive for MPL^W515L^ mutation [42,43].

## 4. CALR

*CALR* is a molecular chaperone protein localizing in the endoplasmic reticulum. In 2013, recurrent mutations in CALR were described as a key MPN driver mutations, which occur in a mutually exclusive manner with *JAK2* and *MPL* mutations [6,7]. Two main mutations account for approximately 80% of all identified *CALR* mutations: a 52-base deletion (del52) (type 1: c.1092_1143del) and 5-base insertion (ins5) (type 2: c.1154_1155insTTGTC). However, more than 50 types of *CALR* mutation have been reported. All of them are base-pair insertions (+2) or deletions (−1) leading to a +1 base-pair frameshift that generates a mutant-specific 36 amino acid sequence in the CALR C-terminus. The mutant-specific C-terminus is found in all *CALR* mutant-expressed proteins and central to the pathogenesis of disease [6,7]. Subsequent studies by different groups demonstrated that the mutant CALR protein causes constitutive activation of MPL and downstream JAK-STAT signaling [44,45,46,47,48,49]. *CALR* alongside *JAK2* and *MPL* mutations are included as major diagnostic criteria for PMF by the World Health Organization (WHO) classification of myeloproliferative neoplasms. *CALR* mutations are associated with better prognosis and lower risk of thrombosis in patients with PMF compared to JAK2 mutations [6,50,51]. However, the favorable prognostic effect of CALR mutations in PMF might be restricted to the type 1 mutant [52].

In 2016, Marty et al. showed that both *CALR^del52^* and *CALR^ins5^* mutants can be expressed in mouse lineage-negative bone marrow (BM) cells using retroviral vectors (pMSCV-IRES-GFP) to induce MPN, with MF developing in *CALR^del52^*-expressing mice [22] (Table 1). *CALR^delex9^* (lacking the entire exon 9) did not induce an MPN phenotype, suggesting that gain of function of the *CALR* mutant C terminus rather than loss of the WT C-terminal sequence drives the MPN phenotype. CALR^del52^ conferred a competitive advantage over WT HSC in vivo and in vitro, while this phenotype was not reported for CALR^ins5^. However, CALR^del52^ and *CALR^ins5^* both amplify the megakaryocyte (MK) lineage. All *CALR* mutant-expressing mice rapidly developed thrombocytosis due to MK hyperplasia. After 6 months, *CALR^del52^*-transduced mice developed MF associated with splenomegaly and a marked osteosclerosis;, however, this was rarely observed in CALR^ins5^. MK progenitors expressing *CALR*^del52^ displayed a hypersensitivity to TPO, exhibited an increased growth in the absence of TPO, and induced MPL activation and JAK/STAT signaling. Interestingly, growth of CALR^del52^ MK progenitors and thromobocytosis was dependent on Mpl expression. *CALR^del52^-Tpo*−/− recipient mice developed ET, suggesting that TPO is not required for in vivo induction of MPN by *CALR*^del52^ [22].

Transgenic [53] or knock-in mice [37,54] expressing human CALR^del52^ mutant protein [37] have also been generated; however, MF was only observed in one model, potentially due to the difference in transgene loci insertion and expression (Table 2). Shide et al. introduced mutant CALR complementary DNA in intron 5 of *Tmigd3* on mouse chromosome 3 [53], driven by the H-2KB promoter and Moloney murine leukemia virus long terminal repeat. These CALR mutant mice develop ET, which is sensitive to ruxolitinib treatment. In contrast, Li et al. generated a conditional mouse knock-in model of the human mutant C-terminus (CALR^del52^) knocked into the mouse Calr gene locus, resulting in mutant CALR expression under the control of the endogenous mouse Calr locus [37]. These heterozygous CALR mutants developed a transplantable ET-like disease with marked thrombocytosis associated with increased and morphologically abnormal MK and increased HSC numbers. Homozygous CALR^del52^ mice developed extreme thrombocytosis accompanied by features of MF, including leukocytosis, reduced hematocrit, splenomegaly, and increased bone marrow reticulin fibrosis. Interestingly, CALR^del52^ HSC did not display a competitive advantage upon transplantation in primary or secondary recipient mice. These data suggest that the expression level of the CALR^del52^ mutant contributes to MF development. More recently, Balligand et al. generated germline Calr^del52^ knock-in mice using Clustered Regularly Interspaced Short Palindromic Repeats-Cas9 (CRISPR/Cas9) [54]. These mice had a transplantable ET phenotype but did not develop MF. Finally, *CALR* sequencing data from patients has shown that CALR^del52^ is more enriched in MF (70%) compared to CALR^ins5^ (13%) [55,56] and that there is evidence of increased susceptibility to MF for CALR^del52^ mutations as compared to CALR^ins5^ mutations [57].

## 5. Other Models of MF

### 5.1. TPO Overexpression

Although overexpression of TPO is not found in patients with MF, two groups initially reported induction of MF by retroviral expression of murine TPO within bone marrow cells [23,24] (Table 1). Very similar phenotypes were observed in these studies, that is, enforced expression of murine TPO in bone marrow transplanted cells leads to fatal MPN with associated MF and osteosclerosis. Interestingly, biphasic disease reminiscent of human MF was observed. During the first 10 weeks, posttransplant mice presented with megakaryocytosis and granulocytosis in both the spleen and bone marrow with erythroblastic hypoplasia in the bone marrow. In the second phase, pancytopenia, a drop in hematopoietic progenitors, osteosclerosis, together with spleen and bone marrow fibrosis were observed.

Using the retroviral TPO overexpression model and genetic fate mapping, Decker et al. investigated the role of Leptin receptor (*Lepr*) expressing MSC in MF development [58]. Cells that were positive for *Lepr* and platelet-derived growth factor receptor (PDGFR) α were the source of fibrosis-driving myofibroblasts and expanded extensively in the bone marrow fibrosis induced by TPO overexpression. Depletion of *PDGFRα* in myofibroblasts or treatment with imatinib, which also targets the *PDGFR* kinase domain, improved the MF phenotype in these mice, resulting in higher BM cellularity and amelioration of BM fibrosis. Although megakaryocyte dysplasia and fibrosis in the spleen was not changed upon *PDGFRα* depletion, the favorable effects on BM fibrosis suggest that targeting *PDGFRα* might be a potential strategy in treating MF.

Kakumitsu et al. generated murine *TPO* transgenic (*TPO* Tg) mice [38] driven by the IgH promoter. Similar to the retroviral system, the authors showed elevated platelets and neutrophils in peripheral blood and increased numbers of megakaryocytes and granulo-myelomonocytic progenitor cells in the bone marrow. Anemia was also observed in *TPO* Tg mice, while erythrocyte progenitors were increased. This suggests a block or a shift in erythroid differentiation toward megakaryocyte/platelet differentiation. *TPO* Tg mice developed MF and osteosclerosis starting at 9 months with progression at 12 months, associated with splenic extramedullary hematopoiesis. It is thought that the local production of *TPO* in the bone marrow and spleen drives the development of fibrosis and osteosclerosis in that model.

### 5.2. Gata-1^low^ Mouse

*Gata-1* is an essential hematopoietic transcription factor regulated by an 8-kb upstream region containing a DNase hypersensitive site or enhancer region. *Gata-1^low^* transgenic mice were generated by replacing this cis-acting enhancer element by a neomycin-resistance cassette using homologous recombination in mouse embryonic stem cells [39]. In the CD1 background, most of mice survived until adulthood but were anemic at birth. These mice present with impaired megakaryocyte maturation and consequent thrombocytopenia. *Gata-1^low^* mice develop slow progressive MF, resembling the development of PMF in patients (Table 2) [59]. They become anemic from 5 months of age and anemia is associated with tear-drop poikilocytes, circulating progenitor cells, and fibrosis in the marrow and in the spleen. Bone marrow from *Gata-1^low^* mutant mice is enriched for osteocalcin, *TGFβ-1*, platelet-derived growth factors (*PDGF*), and vascular endothelial growth factor transcript compare to control mice [60]. Subsequent mechanistic investigation performed in that model identified alteration of *TGFβ-1*, hedgehog, and p53 signaling pathways [61]. Inhibition of TGFβ-1with SB431542 normalized TGFβ-1 signaling and expression of *p53-*related genes. This was associated with restoration of hematopoiesis and normal megakaryocyte development, while the authors observed reduced fibrosis, neovascularization, and osteogenesis in the bone marrow. These data further support a pivotal role of TGFβ-1 in the pathogenesis of MF.

In recent years, Zingariello et al. characterized the Tpo/Mpl axis and downstream Jak/Stat activation in the *Gata-1^low^* mouse model in more detail, finding good agreement with key pathological features that are characteristic for PMF patients [62]. *Tpo* expression was elevated in liver and plasma of Gata-1^low^ mice, and higher expression levels of *Mpl* were detected in lineage –, Sca1+, cKit+ (LSK) cells. Jak2 and Stat5 protein expression was increased in BM and spleen of Gata-1^low^ mice. Treating *Gata-1^low^* mice with ruxolitinib reduced spleen size and Jak2 expression in the spleen but did not have an effect on Jak2/Stat5 levels in the bone marrow or bone marrow cellularity and did not ameliorate BM fibrosis. Interestingly, Zingariello et al. observed poorly developed endoplasmic reticulum with rare polysomes in megakaryocytes of *Gata-1^low^* mice using electron microscopy, supporting their finding of ribosomal deficiencies.

More recently, the *Gata-1^low^* model was used to investigate the *PDGF* system and regulation of *PDGFRβ* in bone marrow fibrosis. Using multispectral imaging, Kramer et al. showed increased *PDGFRβ* expression in bone marrow stromal cells in MF [59]. *PDGF-B* expression was likewise increased but found to be mainly derived from megakaryocytes, supporting the hypothesis that growth factors produced by malignant cells drive bone marrow fibrosis. Furthermore, T-cell protein tyrosine phosphatse (TC-PTP) was identified as a negative regulator of *PDGFβ* in bone marrow fibrosis.

### 5.3. Trisomy 21 Mouse

Trisomy 21 is frequently found as a somatic aberration in myeloid malignancies. Kirsammer et al. observed highly penetrant MPN with modest MF in Ts65Dn mice [40] (Table 2). These mice are trisomic for 104 orthologs of the mouse chromosome 21 (Hsa21). Cellular compartment analysis showed accumulation of stem cells (LSK) and megakaryocytes, together with thrombocytosis, granulocytosis, and anemia. Interestingly, as opposed to many models, splenomegaly was not observed in Ts65Dn mice, despite the bone marrow fibrosis.

### 5.4. Abi-1 Knockout Mouse

Chorzalska et al. recently demonstrated that Abelson interactor 1 (*ABI-1*), a negative regulator of *ABL*, is downregulated in CD34+ cells from PMF patients. Reduced ABI-1 gene expression was observed in granulocytes from PMF patients and patients with MF secondary to PV but not in ET, PV, or MF secondary to ET. Hence, Chorzalska et al. induced conditional deletion of *Abi-1* in mice using the Mx1Cre system [63] (Table 2). Loss of *Abi-1* in the bone marrow of mice resulted in a PMF phenotype featuring leukocytosis, thrombocytosis, anemia, splenomegaly, megakaryocytosis, and fibrosis in the bone marrow. HSC self-renewal was impaired in competitive bone marrow transplant experiments, and Src family kinases, Stat3, and NF-κB signaling were found to be more activated in Abi -deficient mice. Interestingly, there was no increase in Jak2/Stat5 signaling in the BM of *Abi-1* knockout mice. Transducing heterozygous Abi-1 knockout mice with *Mpl^W515L^* accelerated development of the MPN phenotype, showing that loss of *Abi-1* cooperates with mutant *Mpl* to induced MPN. Thus, Abi-1-deficient mice are an interesting new model to interrogate Src, Stat3, and NF-κB signaling in MF.

## 6. Disease Evolution Through Additional Genetic Mutations

### JAK2^VF^-LNK

Upon TPO stimulation of MPL, the adaptor protein *LNK* binds to JAK2 and negatively regulates JAK-STAT signaling [64]. However, *JAK2^VF^* and *LNK* mutations are not necessarily mutually exclusive, as they have been found co-mutated in patients with PMF [65,66]. Interestingly, in a mouse model, LNK loss exacerbates JAK2^VF^-driven MPN and accelerates the development of MF through the potentiation of *JAK2^VF^* signaling [67]. *LNK*-deficient mice exhibit a mild, chronic myeloid leukemia (CML)-like phenotype [68], demonstrating how acquisition of additional mutations can modulate disease progression (Table 3).

## 7. Genetic Lesion in Epigenetic Regulators

Genes encoding epigenetic regulators such enhancer of zeste homologue 2 (*EZH2*), DNA methyl transferase 3A (*DNMT3A*), and additional sex combs-like 1 (*ASXL1*) are among the most frequently mutated genes in patients with MPN [74,75,76]. These mutations usually coexist with one of the three main driver mutations of MPN (i.e., *JAK2*, *CALR*, or *MPL*) [77], and the presence of certain additional mutations (e.g., *ASXL1*) has a powerful, adverse effect on clinical outcomes [78]. Recent publications have now provided evidence of cooperation between driver mutation-associated constitutive activation of JAK-STAT signaling and mutations in epigenetic regulators, as discussed below.

### 7.1. JAK2^VF^ -EZH2

*EZH2*, a histone methyl transferase member of the polycomb repressive complex 2 (PRC2), is a master regulator of chromatin topology that mediates silencing through di- and trimethylation of lysine H3 (H3K27me2/3) [79,80]. Overexpression of Ezh2 induces MPN in mice [81]. Activating mutations in *EZH2* have been observed in malignant B cell lymphomas [82,83]; however, loss of function mutations in *EZH2* are frequently identified in patients with myelodysplastic syndrome (MDS) and MF [84] and loss of function is associated with drug resistance and adverse clinical outcome in hematologic malignancies [85,86,87].

In vivo cooperativity between *Jak2^VF^* and *Ezh2* loss of function was reported by three independent groups [69,70,71], using intercrossed genetically engineered mouse models of *Jak2^VF^* [27,29,36] and *Ezh2* [88,89,90] (Table 3). In two studies, expression of *Jak2^VF^* and the deletion of *Ezh2* were simultaneously induced in hematopoietic stem cells by polyinosinic–polycytidylic acid (pIpC) injection and induction of Mx1=Cre [69,70]. Conversely, Sashida et al. deleted *Ezh2* by tamoxifen injection within secondary hosts transplanted with bone marrow cell from *Jak2^VF^-Ezh*2^flox/flox^-Cre^ERT2^ [71]. These complementary studies demonstrated that coincident induction of *Jak2^VF^* and deletion of *Ezh2* leads to impairment of erythropoiesis and alterations of megakaryopoiesis, highlighted by expansion of megakaryocytic precursors and increase in platelet counts compared to *Jak2^VF^* mice. Moreover, reticulin fibrosis develops in the bone marrow and spleen of these mice. Loss of *Ezh2* enhanced the repopulation capacity of *Jak2^VF^* expressing HSC, and transplantation of bone marrow from *Ezh2*-deleted *Jak2^V^*^F^ resulted in accelerated progression to MF. Mechanistically, *Ezh2* deletion altered PRC2 function and lead to marked decrease H3K27 tri-methylation (H3K27me3) repressive marks in HSPC with associated increase in H3K27 acetylation activation marks (H3K27ac) in *Jak2^VF^* mice. PRC2 targets, such as the oncogenes *Mlf1* and *Pbx3*, and inflammatory regulators such as *S100a8*, *S100a9*, *Ifi27l2a*, *Lin28b Hmga2*, and *TGFβ1* were upregulated. *HMGA2* has been reported to be activated in CD34+ cells from patients with PMF [91,92].

Sashida et al. demonstrated that targeting this epigenetic deregulation using bromodomain inhibition leads to abrogation of MF-initiating cells and is associated with a significant H3K27ac attenuation at the promoter regions of PRC2 target gene in *Jak2^VF^-Ezh2*^-/-^-recipients mice [71]. Also, as previously reported in MF, TNF/NF-κB inflammation and TGFβ-1 signaling were enriched in these mouse models, confirming the important role of these pathways in the development of MF [70].

### 7.2. JAK2^VF^–ASXL1

Mutations in the Additional Sex Combs-Like 1 (*ASXL1*) epigenetic regulator have been found to occur at high frequency in patients with myeloid malignancies (48% of CML, 20% of MDS, 10% of MPN, and 20% of AML) [85,93,94,95,96,97,98,99,100,101,102]. *ASXL1* mutations are associated with poor prognosis and leukemic transformation [103]. Interestingly, these mutations are more common with advanced age and are also found in patients with clonal hematopoiesis of indeterminate potential (CHIP) [104,105,106]. *ASXL1* contains an N-terminal ASX homology (ASXH) domain and a C-terminal plant homeodomain (PHD). The majority of patient-derived *ASXL1* mutations are nonsense or frameshift downstream of the ASXH domain, leading to truncation and loss of the PHD domain [98,100,102,107]. It remains unclear if *ASXL1* confers a gain of function due to expression of a truncated protein or whether a somatic mutation leads to loss of function. Additional studies are required to more accurately understand *ASXL1* mutations in the context of MPN. In mouse models, both loss and Truncated Asxl1 expression leads to altered erythropoiesis [108,109]. Mechanistically, *ASXL1* physically interacts with PRC2 and *ASXL1* silencing is associated with loss of PRC2-mediated gene repression and global loss of H3K27me3 [107]. Recently, Yang et al. described a gain of function mediated by expression of *ASXL1* truncating protein in the pathogenesis of myeloid malignancy [110]. Here they demonstrated that expression of ASXL1^aa1-587^ truncating protein in the hematopoietic system was sufficient to lead to diverse myeloid malignancies such as MDS, MPN, and AML. ASXL1^aa1-587^ expression increases HSC/HSPC functions and increases chromatin accessibility in cKit+ hematopoietic precursors cells. Importantly, they demonstrate that ASXL1^aa1-587^ interacts with BRD4 in bone marrow cells, providing a rationale to treat these malignancies with BET bromodomain inhibitors.

*ASXL1* mutations are found in 34.5% of PMF patients [111]. In line with other epigenetic-related mutations; coincidence of *JAK2^VF^* and *ASXL1* mutations are more frequent in secondary acquired MF (post-PV; 26%) than in PV patients without MF (4%) [72]. Guo et al. crossed *Jak2^VF^* mice [28] with *Asxl1*^+/−^ [112] to generated a *Jak2^VF^-Asxl1*^+/−^ transgenic mice (Table 3). They demonstrated that cooperation of Jak2^VF^ and *Asxl1* heterozygous loss accelerates secondary MF when compared to Jak2^Vf^ and/or Asxl11^+/−^ mice alone. Notably, 12% of *Jak2^VF^-Asxl1*^+/−^mice, but not the littermate controls, progressed to secondary AML at 6–8 months of age. Even at early time points (2–3 months), *Jak2^VF^-Asxl1*^+/−^ mice showed increased white blood cell and neutrophil counts in the peripheral blood when compared with the WT but not with Jak2^VF^ mice, with increased MEPs and CD41+CD61+ megakaryocytic precursors and erythroid precursors in BM and spleen. *Jak2^VF^-Asxl1*^+/−^ mice exhibited splenomegaly with disrupted splenic architecture and prominent megakaryocytes and myeloid precursors. Finally, reticulin staining revealed extensive fibrosis in the BM at the age of 3 months. However, after 8–10 months, *Jak2^VF^-Asxl1*^+/−^ mice display BM failure with anemia and reduced BM cellularity, consistent with progression to advanced MF. More investigation may provide additional information about chromatin landscape and epigenetic changes in this model.

### 7.3. JAK2^VF^–DNMT3A

*DNMT3A* is a de novo DNA methyltransferase that catalyzes the addition of methyl groups into active chromatin in CpG-rich regions, leading to gene inactivation [113,114]. *DNMT3A* mutations are found in up to 22% of AML patients [115]. Interestingly, *DNMT3A* mutations are found at low rates below 5% in primary MPN (PV and ET) but appear to be more frequent in advanced MPN (10–15% of PMF, 15% of secondary MF, and 17% of AML arising from MPN) [115,116,117]. Mutations in *DNMT3A* cluster in the methyltransferase domain, leading to dominant negative function with loss of DNA binding and reduced catalytic activity [118,119]. *Dnmt3a*^-/-^ HSC have enhanced self-renewal capacities and a block in differentiation upon serial transplantation in vivo [120]. This may relate to the observation that *DNMT3A* mutations are frequently found in CHIP [121,122]. In AML, *DNMT3A* mutations mediate resistance to chemotherapy drugs through altering chromatin conformation [123].

In 2018, we reported that Dnmt3a loss and Jak2^VF^ could cooperate to induce MF, using in vivo CRISPR-Cas9 targeting to disrupt Dnmt3a function within Jak2^VF^ LSK [73]. *Jak2^VF^-Dnmt3a*-Cas9 progenitors were transplanted into irradiated recipients and showed a biphasic disease reminiscent of secondary transformation of PV to MF (Table 3). At early time points (8 weeks), Jak2^VF^-Dnmt3a-Cas9 recipients showed high hematocrit, platelet, and white blood cells counts; however, by 32 weeks, mice became pancytopenic with progressive BM failure. Furthermore, a dense fibrocellular infiltrate and BM osteosclerosis developed in *Jak2^VF^-Dnmt3a*-Cas9 recipients, together with disorganized and effaced splenic architecture, dense reticulin fibrosis, and massive splenomegaly. Blood smears from *Jak2^VF^-Dnmt3a*-Cas9 recipients showed severe anemia with left shifted myelopoiesis, anisocytosis, poikilocytosis, and tear-drop erythrocytes, reminiscent of human MF. In this model, myelofibrotic transformation was associated with HSC depletion and the accumulation of multipotent progenitors (MPP). Transcriptional analysis revealed gene expression changes associated with not only HSC identity and function but also proinflammation (TNFα via NF-κB), and these changes were also seen when comparing PMF patients vs. ET, PV, or healthy individuals. Mechanistically, loss of *Dnmt3a* caused PRC2 alteration and increased chromatin accessibility at active enhancers of *Jak2^VF^-Dnmt3a*-Cas9 upregulated genes, including the aforementioned stemness and inflammatory-related pathways such as TNFα/NF-κB signaling. These data demonstrate that Dnmt3a loss of function was sufficient to drive MF through activated TNFα signaling and pro-inflammatory gene expression.

## 8. Signaling Pathway Activation

Heterogeneity in the fibrosis phenotype between driver mutants in *JAK2*, *MPL*, or *CALR* and the type of models (knock-in/retroviral) appears to be related to allele burden/expression level. However, recent studies point out that acquisition of additional mutations (*Asxl1*, *Ezh2*, or *Dnmt3a*) can also alter transcriptional program and can accelerate disease transformation from early stage MPN (PV/ET) toward MF in a scenario where low allele expression of the mutant driver might not be sufficient. It would be interesting to test in *CALR* mutant models if additional mutation such as *Ezh2*, *Dnmt3a*, or *Asxl-1* could accelerate MF transformation (in CALR^del52^) or MF incidence (in CALR^ins5^).

## 9. Using Mouse Models to Validate Preclinical Therapeutic Strategies for MF

Murine models of MF provide excellent opportunities to develop and validate preclinical therapeutic strategies for patients with MF. Inflammation has been a key and recurrent feature of MF development, and strategies targeting cytokine inflammation hold great promise clinically. Kleppe et al. further demonstrated that ruxolitinib treatment normalized cytokine expression and that *JAK2* inhibition reduced cytokine production from both normal and mutant cells in vivo [9]. Among the aberrantly activated networks, they identified TNF/NF-κB inflammatory signaling as a key pathway activated in both malignant and nonmalignant cells and as a common pathway in MF progression, thus providing insights into the molecular basis of MPN-associated inflammation and a therapeutic approach to target aberrant inflammatory signaling in MPN.

In an alternative approach to JAK2 inhibition, Yue et al. used the *MPL*^W515L^ model to target transforming growth factor β-1 (TGFβ-1) signaling [124]. Administration of galunisertib, an inhibitor of TGF-β receptor I kinase (ALK5), decreased collagen deposition by MSC in the bone marrow and improved MF in the MPL^W515L^ model. However, ALK5 inhibition did not have an effect on blood cell counts or splenomegaly. Yue and colleagues successfully reproduced the beneficial effects of ALK5 inhibition on BM fibrosis in the transgenic *JAK2^VF^* mice generated by Xing et al., indicating that targeting the TGFβ-1 axis might be a promising treatment strategy [28]. These findings are also supported from work using TPO overexpression, as TGFβ-1 is required for fibrosis development in that model. TPO expression in *Tgfb1* null mice donor cells could not recapitulate MF physiopathology [125]. Complementary works demonstrated later that thrombospondin (Tsp) is not the major activator of TGF-β1 [126] and that osteoprotegerin (Opg) is required for osteosclerotic transformation in this model [127].

Interestingly, Verstovsek et al. have reported that monocyte-derived fibrocytes are abundant in primary myelofibrosis and produce collagen [128]. In this study, immunodeficient mice transplanted with bone marrow cells from patients with myelofibrosis developed a lethal myelofibrosis-like phenotype and, importantly, that treatment with the fibrocyte inhibitor, serum amyloid P, slowed the development of fibrosis.

More recently, Schneider and colleagues demonstrated that TPO transduced cells promote the outgrowth of Gli1+ MSC that expand into fibrosis in the bone marrow of recipient mice [129]. Mechanistically, the authors elegantly demonstrated that hematopoietic cells overexpressing *TPO* induced expression of profibrotic factors such as *Cxcl4*, *Endothelin 1*, and *MMP9* [130] by stromal cells. Release of chemokine Cxcl4 by hematopoietic and stromal cells is necessary and sufficient to attract Gli1+ stromal cells and to induce their differentiation into myofibroblastic cells. Gli1 antagonist 61 (GANT61), a small-molecule inhibitor of fibrosis [131,132] with the potential to normalize production of fibrotic factors, significantly reduced the number of Gli1+-myofibroblasts and malignant hematopoietic cells and abolished the development of reticulin fibrosis. This mechanism is relevant to patients, emphasized by the finding that *Gli1+* MSC also expand in human MF and are sensitive to GANT61 inhibition [130]. Thus, this model was able to provide a rationale for targeting the GLI1 pathway in MF.

## 10. Discussion

The mechanisms leading to MF converge on the amplification and modification of signaling pathways, i.e., JAK2 signaling via JAK/STAT, IFNγ response, INFα response, TGFβ-1, p53, and hedgehog and TNFα signaling via NF-κB [8,69,70,73,129,133]. There are many similarities across the MF models including cytokine dysregulation, inflammatory pathway activation, megakaryocytic morphologic abnormalities, and enhancer reprogramming, leading to HSC dysfunction. In the constitutively active *MPL* mutant (*MPL^W515L^*), JAK-STAT activation (STAT3) was required in malignant and nonmalignant hematopoietic cells, and this identified six cytokines (Il6, IL10, IL12, TNFα, CCL2, and CCL5) aberrantly produced that were also found to be increased in granulocyte from patients with MF. Importantly, ruxolitinib treatment normalized cytokine expression and reduced cytokine production from both normal and mutant cells in vivo [9]. Interestingly, highly sensitive protein studies suggest that cytokine levels remain markedly abnormal in MF, even in the presence of JAK2 inhibition [134].

Kleppe et al. showed that, both in the retroviral *MPL^W515L^* model and *Jak2^V^*^F^ knock-in mice, MF-related inflammation is associated with alterations of chromatin regulatory element such as enhancer and promoter regions in the MF clone, also seen in other studies [72,73]. These findings suggest that different genetic lesions may alter similar pathways within hematopoietic progenitors in MF.

TNFα via the NF-κB inflammatory signaling pathway was also identified as central to pathophysiology in two additional MF models: *Jak2^VF^-Ezh2*^-/-^ [70] and *Jak2^VF^-Dnmt3a* [73]. However, in those two MF models, TGFβ-1 signaling was only enriched in *Jak2^VF^-Ezh2*^-/-^ recipients, perhaps explaining the absence of megakaryocytosis in *Jak2^VF^-Dnmt3a*-Cas9 mice [61]. It cannot be excluded that this difference is due to disease kinetics as bone marrow observation and transcriptional analysis were not performed under strictly comparable condition across studies. Interestingly, elevated TGFβ-1 was found in plasma from *JAK2^VF^* homozygous but not *JAK2^VF^* heterozygous patients compared to healthy controls [61].

MF is a chemo-refractory disease associated with a poor clinical prognosis. So far, the only curative treatment is allogeneic stem cell transplantation which is only available to a minority of patients. Conventional treatment is mainly aimed at controlling symptoms and complications. For many years, the cytoreductive agent hydroxyurea was the therapy of choice for MF splenomegaly. Here, the overall response is 40% and the median duration is 13.2 months [135]. Oral or leg ulcers and progressive cytopenias are related toxicities of hydroxyurea and may be dose limiting. More recently, JAK2 inhibitors have become the standard of care in patients with high or intermediate risk MF [136,137,138]. JAK inhibitors such as ruxolitinib and fedratinib can control the symptoms of MF, in particular splenomegaly, but MPN cells are not cleared by these drugs, perhaps due to the inherent resistance of stem cell populations [139] or by persistent cross activation of JAK-STAT signaling [140]. In the latter example, JAK inhibitor persistence is reversible and is associated with site-specific changes in the chromatin state, consistent with an epigenetic mechanism by which MPN cells evade JAK kinase inhibition.

The findings from murine models of MF give some direction to new possibilities in the treatment of MF. Kleppe et al. have shown that BET Inhibition with (JQ1) attenuates NF-κB transactivation in vivo and that combined ruxolitinib/JQ1 delays JAK inhibitor persistence and shows efficacy against MPN cells [8]. These findings are supported by the work of Sashida et al., who demonstrated that bromodomain inhibition leads to abrogation of MF-initiating cells and is associated with H3K27ac attenuation at the promoter regions of PRC2 target genes in a *Jak2^VF^-Ezh2*^-/-^ model [71]. Together, these studies provide strong evidence and rationale for targeting the bromodomain protein in MF. Despite their strong efficiency in MF, the use of BET inhibitor molecules has initially been limited due to dose-limiting toxicities including thrombocytopenia, fatigue, nausea, vomiting, and diarrhea [141]. However, use of CPI-0610 alone or in combination with ruxolitinib showed encouraging results with good tolerability [142,143].

Altered chromatin landscape has been described in a number of mouse models [8,69,70,71,73]. Loss of H3K27me3 repressive marks at chromatin regulatory element (promoter or enhancer) controlling inflammation-related genes appears to be a common mechanism driving MF pathology, also implicating PRC2 as a key element in MF pathology. PRC2 is essential for controlling hematopoietic cell differentiation, shapes chromatin regulatory components by turning off stemness genes, and facilitates a normal differentiation program. Chromatin landscape is thoroughly regulated during hematopoiesis [144]. Thus, alteration of PRC2 function leads to topological changes in chromatin, failure to repress developmental enhancers, abnormal stem cell differentiation, and inflammatory programs in the context of MF.

## 11. Conclusions

In MPN, progression to MF represents a high-risk clinical scenario and murine models of disease demonstrate the important role that MPN phenotypic driver gene dosage and the acquisition of additional genetic lesions play in this process. Mechanistically, pathways to MF appear to converge on epigenetic deregulation and pro-inflammatory signaling pathway activation, identifying these pathways as tractable therapeutic opportunities. Moving forward, translational clinical trials will be needed to address the optimal ways to target these pathways in patients with MF to improve long-term outcomes, survival, and potentially even cure.

## Figures and Tables

**Table 1:** Models of myelofibrosis generated by retroviral overexpression and bone marrow transplantation in mice. ET: essential thrombocythemia, MF: myelofibrosis, TPO: thrombopoietin, PV: polycythemia vera.

Model	Strain	Vector	Phenotype	References
Jak2^VF^	C57Bl/6	MEGIX	PV with progression to MF	[18]
Jak2^VF^	C57Bl/6 and Balb/c	MSCV	PV, progression to MF was only observed in Balb/c mice	[19]
Mpl^W515L^	Balb/C	MSCV	Features of ET with progression to MF	[21]
Calr^del52^	C57Bl/6	MSCV	ET with progression to MF	[22]
TPO	BDF1	MSCV	ET with progression to MF	[23]
TPO	C57Bl/6	MPZen2	ET with progression to MF	[24]

**Table 2:** Transgenic and knock-in mouse models of myelofibrosis. BMT: bone marrow transplant, ET: essential thrombocythemia, MF: myelofibrosis, PMF: primary myelofibrosis, TPO: thrombopoietin, PV: polycythemia vera.

	Model	Type	Strain	Locus	Activation	Phenotype	References
Driver mutations	Jak2^VF^	Transgenic	BDF1	Line 1: *Dcc* intron 12 on chr18 Line 2: between *Mef2a* and *Lrrc28* on chr7, expression under H-2Kb promoter	Constitutive	Line 1: about 50% PV or ET, 50% no clear phenotype Line 2: PMF	[27]
	Jak2^VF^	Transgenic	C57BL/6 XDBA	Locus n/a, expression under *Vav* promoter	Conditional	ET, PV or PMF	[28]
	JAK2^VF^	Transgenic	C57Bl/6	Human JAK2^VF^, expression under human *JAK2* promoter	Conditional (VavCre), inducible (Mx1Cre)	VavCre: ET with progression to MF Mx1Cre: PV with progression to MF	[36]
	Jak2^VF^	Knock-in	129Sv C57Bl/6	Endogenous *Jak2* locus on chr19	Conditional, inducible (Mx1Cre)	PV with progression to MF in heterozygous, stronger phenotype in homozygous mice	[29]
	Jak2^VF^	Knock-in	129Sv C57Bl/6	Endogenous *Jak2* locus on chr19	Constitutive	PV with progression to MF in heterozygous mice	[31]
	Jak2^VF^	Knock-in	C57Bl/6	Endogenous *Jak2* locus on chr19	Conditional, (E2ACre)	PV, homozygosity embryonically lethal, MF only in secondary recipients	[32]
	JAK2^VF^	Knock-in	C57Bl/6	Human JAK2^VF^ in endogenous *Jak2* locus on chr19	Conditional, inducible (Mx1Cre)	ET, PV and one mouse with MF in BMT	[30]
	JAK2^VF^	Knock-in	C57Bl/6	Endogenous *Jak2* locus on chr19	Conditional (VavCre)	PV with progression to MF	[33]
	Calr^del52^	Knock-in	C57Bl/6	Endogenous *Calr* locus on chr8	Conditional, inducible (Mx1Cre)	ET in heterozygous, ET with progression to MF in homozygous mice	[37]
Other models	TPO	Transgenic	BDF1	Locus n/a, driven by the IgH promoter	Constitutive	Features of ET with progression to MF	[38]
	Gata-1^low^	Knock-out (upstream promotor region of *Gata1*)	C57Bl/6CD1	Endogenous promotor region on chrX	Constitutive	>90% mortality in C57Bl/6, normal life span with develop-ment of PMF in CD1	[39]
	Trisomy 21 (Ts65Dn)	Chromosomal translocation	C57Bl/6	Chr16 (harboring 2/3 of human chr21 genes) translocation to chr17	Constitutive	Down syndrome, features of ET with progression to MF	[40]

**Table 3:** Mouse models of myelofibrosis targeting multiple genes. BMT: bone marrow transplant, ET: essential thrombocythemia, MF: myelofibrosis, PMF: primary myelofibrosis, TPO: thrombopoietin, PV: polycythemia vera.

Model	Type	Strain	Jak2^VF^ model (locus)	Activation	Phenotype	References
Jak2^VF^-LNK	Retroviral overexpression (MIG)/BMT (Jak2^VF^) Knockout (LNK)	C57BL6/J	/	Constitutive (LNK knockout)	PV with progression to MF is accelerated in double mutant	[67]
JAK2^VF^-Ezh2	Transgenic (Jak2^VF^) Knockout (Ezh2)	C57BL6/J	Tiedt et al. JAK2^VF^ Jak2^VF^ mice (human JAK2^VF^, expression under human *JAK2* promoter)	Conditional, inducible (Mx1Cre, SclCre^ER^)	ET with progression to MF is accelerated in double mutant	[69]
Jak2^VF^-Ezh2	Knock-in (Jak2^VF^) Knockout (Ezh2)	C57Bl/6	Akada et al. 2010 Jak2^VF^ mice (endogenous *Jak2* locus on Chr19)	Conditional, inducible (Mx1Cre)	PV (JAK2^VF^) shifts towards ET in combined model with rapid progression to MF	[70]
Jak2^VF^-Ezh2	Transgenic (Jak2^VF^) Knockout (Ezh2) BMT	C57Bl/6	Shide et al. 2008 Jak2^VF^ mice (line 1 chr18, line 2 chr7, expression under H-2Kb promoter)	Conditional (Cre-ERT2)	MPN features Ezh2-/- with rapid progression to MF	[71]
Jak2^VF^-Asxl1	Transgenic (Jak2^VF^) Knockout (Asxl1)	C57BL/6 XDBA	Xing et al. 2008 Jak2^VF^ mice (locus n/a, expression under *Vav* promoter)	Conditional	ET, PV, or PMF with accelerated MF in combined model	[72]
Jak2^VF^-Dnmt3a	Knock-in (Jak2^VF^) Knockout (Dnmt3a)	C57Bl/6	Mullally et al. 2010 Jak2^VF^ mice (endogenous *Jak2* locus on Chr19)	Conditional	PV with progression to MF in combined model (MF was absent in JAK2^VF^ model)	[73]

## References

[1] James C., Ugo V., Le Couedic J.P., Staerk J., Delhommeau F., Lacout C., Garcon L., Raslova H., Berger R., Bennaceur-Griscelli A. (2005). A unique clonal JAK2 mutation leading to constitutive signalling causes polycythaemia vera. Nature.

[2] Lu X., Huang L.J., Lodish H.F. (2008). Dimerization by a cytokine receptor is necessary for constitutive activation of JAK2V617F. J. Biol. Chem..

[3] Mesa R.A., Verstovsek S., Cervantes F., Barosi G., Reilly J.T., Dupriez B., Levine R., Le Bousse-Kerdiles M.C., Wadleigh M., Campbell P.J. (2007). Primary myelofibrosis (PMF), post polycythemia vera myelofibrosis (post-PV MF), post essential thrombocythemia myelofibrosis (post-ET MF), blast phase PMF (PMF-BP): Consensus on terminology by the international working group for myelofibrosis research and treatment (IWG-MRT). Leuk. Res..

[4] Mehta J., Wang H., Iqbal S.U., Mesa R. (2014). Epidemiology of myeloproliferative neoplasms in the United States. Leuk. Lymphoma.

[5] Tefferi A. (2011). Primary myelofibrosis: 2012 update on diagnosis, risk stratification, and management. Am. J. Hematol..

[6] Klampfl T., Gisslinger H., Harutyunyan A.S., Nivarthi H., Rumi E., Milosevic J.D., Them N.C., Berg T., Gisslinger B., Pietra D. (2013). Somatic mutations of calreticulin in myeloproliferative neoplasms. N. Engl. J. Med..

[7] Nangalia J., Massie C.E., Baxter E.J., Nice F.L., Gundem G., Wedge D.C., Avezov E., Li J., Kollmann K., Kent D.G. (2013). Somatic CALR mutations in myeloproliferative neoplasms with nonmutated JAK2. N. Engl. J. Med..

[8] Kleppe M., Koche R., Zou L., Van Galen P., Hill C.E., Dong L., De Groote S., Papalexi E., Hanasoge Somasundara A.V., Cordner K. (2018). Dual Targeting of Oncogenic Activation and Inflammatory Signaling Increases Therapeutic Efficacy in Myeloproliferative Neoplasms. Cancer Cell.

[9] Kleppe M., Kwak M., Koppikar P., Riester M., Keller M., Bastian L., Hricik T., Bhagwat N., McKenney A.S., Papalexi E. (2015). JAK-STAT pathway activation in malignant and nonmalignant cells contributes to MPN pathogenesis and therapeutic response. Cancer Discov..

[10] Yan D., Hutchison R.E., Mohi G. (2012). Critical requirement for Stat5 in a mouse model of polycythemia vera. Blood.

[11] Schneider R.K., Ziegler S., Leisten I., Ferreira M.S., Schumacher A., Rath B., Fahrenkamp D., Muller-Newen G., Crysandt M., Wilop S. (2014). Activated fibronectin-secretory phenotype of mesenchymal stromal cells in pre-fibrotic myeloproliferative neoplasms. J. Hematol. Oncol..

[12] Rawlings J.S., Rosler K.M., Harrison D.A. (2004). The JAK/STAT signaling pathway. J. Cell Sci..

[13] Baxter E.J., Scott L.M., Campbell P.J., East C., Fourouclas N., Swanton S., Vassiliou G.S., Bench A.J., Boyd E.M., Curtin N. (2005). Acquired mutation of the tyrosine kinase JAK2 in human myeloproliferative disorders. Lancet.

[14] Levine R.L., Wadleigh M., Cools J., Ebert B.L., Wernig G., Huntly B.J., Boggon T.J., Wlodarska I., Clark J.J., Moore S. (2005). Activating mutation in the tyrosine kinase JAK2 in polycythemia vera, essential thrombocythemia, and myeloid metaplasia with myelofibrosis. Cancer Cell.

[15] Jones A.V., Kreil S., Zoi K., Waghorn K., Curtis C., Zhang L., Score J., Seear R., Chase A.J., Grand F.H. (2005). Widespread occurrence of the JAK2 V617F mutation in chronic myeloproliferative disorders. Blood.

[16] Kralovics R., Passamonti F., Buser A.S., Teo S.S., Tiedt R., Passweg J.R., Tichelli A., Cazzola M., Skoda R.C. (2005). A gain-of-function mutation of JAK2 in myeloproliferative disorders. N. Engl. J. Med..

[17] Bumm T.G., Elsea C., Corbin A.S., Loriaux M., Sherbenou D., Wood L., Deininger J., Silver R.T., Druker B.J., Deininger M.W. (2006). Characterization of murine JAK2V617F-positive myeloproliferative disease. Cancer Res..

[18] Lacout C., Pisani D.F., Tulliez M., Gachelin F.M., Vainchenker W., Villeval J.L. (2006). JAK2V617F expression in murine hematopoietic cells leads to MPD mimicking human PV with secondary myelofibrosis. Blood.

[19] Wernig G., Mercher T., Okabe R., Levine R.L., Lee B.H., Gilliland D.G. (2006). Expression of Jak2V617F causes a polycythemia vera-like disease with associated myelofibrosis in a murine bone marrow transplant model. Blood.

[20] Zaleskas V.M., Krause D.S., Lazarides K., Patel N., Hu Y., Li S., Van Etten R.A. (2006). Molecular pathogenesis and therapy of polycythemia induced in mice by JAK2 V617F. PLoS ONE.

[21] Pikman Y., Lee B.H., Mercher T., McDowell E., Ebert B.L., Gozo M., Cuker A., Wernig G., Moore S., Galinsky I. (2006). MPLW515L is a novel somatic activating mutation in myelofibrosis with myeloid metaplasia. PLoS Med.

[22] Marty C., Pecquet C., Nivarthi H., El-Khoury M., Chachoua I., Tulliez M., Villeval J.L., Raslova H., Kralovics R., Constantinescu S.N. (2016). Calreticulin mutants in mice induce an MPL-dependent thrombocytosis with frequent progression to myelofibrosis. Blood.

[23] Yan X.Q., Lacey D., Fletcher F., Hartley C., McElroy P., Sun Y., Xia M., Mu S., Saris C., Hill D. (1995). Chronic exposure to retroviral vector encoded MGDF (mpl-ligand) induces lineage-specific growth and differentiation of megakaryocytes in mice. Blood.

[24] Villeval J.L., Cohen-Solal K., Tulliez M., Giraudier S., Guichard J., Burstein S.A., Cramer E.M., Vainchenker W., Wendling F. (1997). High thrombopoietin production by hematopoietic cells induces a fatal myeloproliferative syndrome in mice. Blood.

[25] Li J., Kent D.G., Chen E., Green A.R. (2011). Mouse models of myeloproliferative neoplasms: JAK of all grades. Dis. Model Mech..

[26] Mullally A., Lane S.W., Brumme K., Ebert B.L. (2012). Myeloproliferative neoplasm animal models. Hematology/oncology clinics of North America.

[27] Shide K., Shimoda H.K., Kumano T., Karube K., Kameda T., Takenaka K., Oku S., Abe H., Katayose K.S., Kubuki Y. (2008). Development of ET, primary myelofibrosis and PV in mice expressing JAK2 V617F. Leukemia.

[28] Xing S., Wanting T.H., Zhao W., Ma J., Wang S., Xu X., Li Q., Fu X., Xu M., Zhao Z.J. (2008). Transgenic expression of JAK2V617F causes myeloproliferative disorders in mice. Blood.

[29] Akada H., Yan D., Zou H., Fiering S., Hutchison R.E., Mohi M.G. (2010). Conditional expression of heterozygous or homozygous Jak2V617F from its endogenous promoter induces a polycythemia vera-like disease. Blood.

[30] Li J., Spensberger D., Ahn J.S., Anand S., Beer P.A., Ghevaert C., Chen E., Forrai A., Scott L.M., Ferreira R. (2010). JAK2 V617F impairs hematopoietic stem cell function in a conditional knock-in mouse model of JAK2 V617F-positive essential thrombocythemia. Blood.

[31] Marty C., Lacout C., Martin A., Hasan S., Jacquot S., Birling M.C., Vainchenker W., Villeval J.L. (2010). Myeloproliferative neoplasm induced by constitutive expression of JAK2V617F in knock-in mice. Blood.

[32] Mullally A., Lane S.W., Ball B., Megerdichian C., Okabe R., Al-Shahrour F., Paktinat M., Haydu J.E., Housman E., Lord A.M. (2010). Physiological Jak2V617F expression causes a lethal myeloproliferative neoplasm with differential effects on hematopoietic stem and progenitor cells. Cancer Cell.

[33] Hasan S., Lacout C., Marty C., Cuingnet M., Solary E., Vainchenker W., Villeval J.L. (2013). JAK2V617F expression in mice amplifies early hematopoietic cells and gives them a competitive advantage that is hampered by IFNalpha. Blood.

[34] Mansier O., Kilani B., Guitart A.V., Guy A., Gourdou-Latyszenok V., Marty C., Parrens M., Plo I., Vainchenker W., James C. (2019). Description of a knock-in mouse model of JAK2V617F MPN emerging from a minority of mutated hematopoietic stem cells. Blood.

[35] Mullally A., Poveromo L., Schneider R.K., Al-Shahrour F., Lane S.W., Ebert B.L. (2012). Distinct roles for long-term hematopoietic stem cells and erythroid precursor cells in a murine model of Jak2V617F-mediated polycythemia vera. Blood.

[36] Tiedt R., Hao-Shen H., Sobas M.A., Looser R., Dirnhofer S., Schwaller J., Skoda R.C. (2008). Ratio of mutant JAK2-V617F to wild-type Jak2 determines the MPD phenotypes in transgenic mice. Blood.

[37] Li J., Prins D., Park H.J., Grinfeld J., Gonzalez-Arias C., Loughran S., Dovey O.M., Klampfl T., Bennett C., Hamilton T.L. (2018). Mutant calreticulin knockin mice develop thrombocytosis and myelofibrosis without a stem cell self-renewal advantage. Blood.

[38] Kakumitsu H., Kamezaki K., Shimoda K., Karube K., Haro T., Numata A., Shide K., Matsuda T., Oshima K., Harada M. (2005). Transgenic mice overexpressing murine thrombopoietin develop myelofibrosis and osteosclerosis. Leuk. Res..

[39] McDevitt M.A., Shivdasani R.A., Fujiwara Y., Yang H., Orkin S.H. (1997). A “knockdown” mutation created by cis-element gene targeting reveals the dependence of erythroid cell maturation on the level of transcription factor GATA-1. Proc. Natl. Acad. Sci. USA.

[40] Kirsammer G., Jilani S., Liu H., Davis E., Gurbuxani S., Le Beau M.M., Crispino J.D. (2008). Highly penetrant myeloproliferative disease in the Ts65Dn mouse model of Down syndrome. Blood.

[41] Pardanani A.D., Levine R.L., Lasho T., Pikman Y., Mesa R.A., Wadleigh M., Steensma D.P., Elliott M.A., Wolanskyj A.P., Hogan W.J. (2006). MPL515 mutations in myeloproliferative and other myeloid disorders: A study of 1182 patients. Blood.

[42] Koppikar P., Abdel-Wahab O., Hedvat C., Marubayashi S., Patel J., Goel A., Kucine N., Gardner J.R., Combs A.P., Vaddi K. (2010). Efficacy of the JAK2 inhibitor INCB16562 in a murine model of MPLW515L-induced thrombocytosis and myelofibrosis. Blood.

[43] Wernig G., Kharas M.G., Mullally A., Leeman D.S., Okabe R., George T., Clary D.O., Gilliland D.G. (2012). EXEL-8232, a small-molecule JAK2 inhibitor, effectively treats thrombocytosis and extramedullary hematopoiesis in a murine model of myeloproliferative neoplasm induced by MPLW515L. Leukemia.

[44] Araki M., Yang Y., Masubuchi N., Hironaka Y., Takei H., Morishita S., Mizukami Y., Kan S., Shirane S., Edahiro Y. (2016). Activation of the thrombopoietin receptor by mutant calreticulin in CALR-mutant myeloproliferative neoplasms. Blood.

[45] Chachoua I., Pecquet C., El-Khoury M., Nivarthi H., Albu R.I., Marty C., Gryshkova V., Defour J.P., Vertenoeil G., Ngo A. (2016). Thrombopoietin receptor activation by myeloproliferative neoplasm associated calreticulin mutants. Blood.

[46] Elf S., Abdelfattah N.S., Chen E., Perales-Paton J., Rosen E.A., Ko A., Peisker F., Florescu N., Giannini S., Wolach O. (2016). Mutant Calreticulin Requires Both Its Mutant C-terminus and the Thrombopoietin Receptor for Oncogenic Transformation. Cancer Discov..

[47] Imai M., Araki M., Komatsu N. (2017). Somatic mutations of calreticulin in myeloproliferative neoplasms. Int. J. Hematol..

[48] Pecquet C., Chachoua I., Roy A., Balligand T., Vertenoeil G., Leroy E., Albu R.I., Defour J.P., Nivarthi H., Hug E. (2019). Calreticulin mutants as oncogenic rogue chaperones for TpoR and traffic-defective pathogenic TpoR mutants. Blood.

[49] Nivarthi H., Chen D., Cleary C., Kubesova B., Jager R., Bogner E., Marty C., Pecquet C., Vainchenker W., Constantinescu S.N. (2016). Thrombopoietin receptor is required for the oncogenic function of CALR mutants. Leukemia.

[50] Finazzi M.C., Carobbio A., Cervantes F., Isola I.M., Vannucchi A.M., Guglielmelli P., Rambaldi A., Finazzi G., Barosi G., Barbui T. (2015). CALR mutation, MPL mutation and triple negativity identify patients with the lowest vascular risk in primary myelofibrosis. Leukemia.

[51] Tefferi A., Guglielmelli P., Lasho T.L., Rotunno G., Finke C., Mannarelli C., Belachew A.A., Pancrazzi A., Wassie E.A., Ketterling R.P. (2014). CALR and ASXL1 mutations-based molecular prognostication in primary myelofibrosis: An international study of 570 patients. Leukemia.

[52] Tefferi A., Lasho T.L., Tischer A., Wassie E.A., Finke C.M., Belachew A.A., Ketterling R.P., Hanson C.A., Pardanani A.D. (2014). The prognostic advantage of calreticulin mutations in myelofibrosis might be confined to type 1 or type 1-like CALR variants. Blood.

[53] Shide K., Kameda T., Yamaji T., Sekine M., Inada N., Kamiunten A., Akizuki K., Nakamura K., Hidaka T., Kubuki Y. (2017). Calreticulin mutant mice develop essential thrombocythemia that is ameliorated by the JAK inhibitor ruxolitinib. Leukemia.

[54] Balligand T., Achouri Y., Pecquet C., Gaudray G., Colau D., Hug E., Rahmani Y., Stroobant V., Plo I., Vainchenker W. (2020). Knock-in of murine Calr del52 induces essential thrombocythemia with slow-rising dominance in mice and reveals key role of Calr exon 9 in cardiac development. Leukemia.

[55] Cabagnols X., Defour J.P., Ugo V., Ianotto J.C., Mossuz P., Mondet J., Girodon F., Alexandre J.H., Mansier O., Viallard J.F. (2015). Differential association of calreticulin type 1 and type 2 mutations with myelofibrosis and essential thrombocytemia: Relevance for disease evolution. Leukemia.

[56] Tefferi A., Wassie E.A., Guglielmelli P., Gangat N., Belachew A.A., Lasho T.L., Finke C., Ketterling R.P., Hanson C.A., Pardanani A. (2014). Type 1 versus Type 2 calreticulin mutations in essential thrombocythemia: A collaborative study of 1027 patients. Am. J. Hematol..

[57] Pietra D., Rumi E., Ferretti V.V., Di Buduo C.A., Milanesi C., Cavalloni C., Sant’Antonio E., Abbonante V., Moccia F., Casetti I.C. (2016). Differential clinical effects of different mutation subtypes in CALR-mutant myeloproliferative neoplasms. Leukemia.

[58] Decker M., Martinez-Morentin L., Wang G., Lee Y., Liu Q., Leslie J., Ding L. (2017). Leptin-receptor-expressing bone marrow stromal cells are myofibroblasts in primary myelofibrosis. Nat. Cell Biol..

[59] Kramer F., Dernedde J., Mezheyeuski A., Tauber R., Micke P., Kappert K. (2020). Platelet-derived growth factor receptor beta activation and regulation in murine myelofibrosis. Haematologica.

[60] Vannucchi A.M., Bianchi L., Cellai C., Paoletti F., Rana R.A., Lorenzini R., Migliaccio G., Migliaccio A.R. (2002). Development of myelofibrosis in mice genetically impaired for GATA-1 expression (GATA-1(low) mice). Blood.

[61] Zingariello M., Martelli F., Ciaffoni F., Masiello F., Ghinassi B., D’Amore E., Massa M., Barosi G., Sancillo L., Li X. (2013). Characterization of the TGF-beta1 signaling abnormalities in the Gata1low mouse model of myelofibrosis. Blood.

[62] Zingariello M., Sancillo L., Martelli F., Ciaffoni F., Marra M., Varricchio L., Rana R.A., Zhao C., Crispino J.D., Migliaccio A.R. (2017). The thrombopoietin/MPL axis is activated in the Gata1(low) mouse model of myelofibrosis and is associated with a defective RPS14 signature. Blood Cancer J..

[63] Chorzalska A., Morgan J., Ahsan N., Treaba D.O., Olszewski A.J., Petersen M., Kingston N., Cheng Y., Lombardo K., Schorl C. (2018). Bone marrow-specific loss of ABI1 induces myeloproliferative neoplasm with features resembling human myelofibrosis. Blood.

[64] Bersenev A., Wu C., Balcerek J., Tong W. (2008). Lnk controls mouse hematopoietic stem cell self-renewal and quiescence through direct interactions with JAK2. J. Clin. Investig..

[65] Lasho T.L., Tefferi A., Finke C., Pardanani A. (2011). Clonal hierarchy and allelic mutation segregation in a myelofibrosis patient with two distinct LNK mutations. Leukemia.

[66] Pardanani A., Lasho T., Finke C., Oh S.T., Gotlib J., Tefferi A. (2010). LNK mutation studies in blast-phase myeloproliferative neoplasms, and in chronic-phase disease with TET2, IDH, JAK2 or MPL mutations. Leukemia.

[67] Bersenev A., Wu C., Balcerek J., Jing J., Kundu M., Blobel G.A., Chikwava K.R., Tong W. (2010). Lnk constrains myeloproliferative diseases in mice. J Clin Invest.

[68] Velazquez L., Cheng A.M., Fleming H.E., Furlonger C., Vesely S., Bernstein A., Paige C.J., Pawson T. (2002). Cytokine signaling and hematopoietic homeostasis are disrupted in Lnk-deficient mice. J. Exp. Med..

[69] Shimizu T., Kubovcakova L., Nienhold R., Zmajkovic J., Meyer S.C., Hao-Shen H., Geier F., Dirnhofer S., Guglielmelli P., Vannucchi A.M. (2016). Loss of Ezh2 synergizes with JAK2-V617F in initiating myeloproliferative neoplasms and promoting myelofibrosis. J. Exp. Med..

[70] Yang Y., Akada H., Nath D., Hutchison R.E., Mohi G. (2016). Loss of Ezh2 cooperates with Jak2V617F in the development of myelofibrosis in a mouse model of myeloproliferative neoplasm. Blood.

[71] Sashida G., Wang C., Tomioka T., Oshima M., Aoyama K., Kanai A., Mochizuki-Kashio M., Harada H., Shimoda K., Iwama A. (2016). The loss of Ezh2 drives the pathogenesis of myelofibrosis and sensitizes tumor-initiating cells to bromodomain inhibition. J Exp Med.

[72] Guo Y., Zhou Y., Yamatomo S., Yang H., Zhang P., Chen S., Nimer S.D., Zhao Z.J., Xu M., Bai J. (2019). ASXL1 alteration cooperates with JAK2V617F to accelerate myelofibrosis. Leukemia.

[73] Jacquelin S., Straube J., Cooper L., Vu T., Song A., Bywater M., Baxter E., Heidecker M., Wackrow B., Porter A. (2018). Jak2V617F and Dnmt3a loss cooperate to induce myelofibrosis through activated enhancer-driven inflammation. Blood.

[74] Abdel-Wahab O., Pardanani A., Patel J., Wadleigh M., Lasho T., Heguy A., Beran M., Gilliland D.G., Levine R.L., Tefferi A. (2011). Concomitant analysis of EZH2 and ASXL1 mutations in myelofibrosis, chronic myelomonocytic leukemia and blast-phase myeloproliferative neoplasms. Leukemia.

[75] Abdel-Wahab O., Patel J., Levine R.L. (2011). Clinical implications of novel mutations in epigenetic modifiers in AML. Hematol. Oncol. Clin. N. Am..

[76] Vainchenker W., Delhommeau F., Constantinescu S.N., Bernard O.A. (2011). New mutations and pathogenesis of myeloproliferative neoplasms. Blood.

[77] Lundberg P., Karow A., Nienhold R., Looser R., Hao-Shen H., Nissen I., Girsberger S., Lehmann T., Passweg J., Stern M. (2014). Clonal evolution and clinical correlates of somatic mutations in myeloproliferative neoplasms. Blood.

[78] Tefferi A., Lasho T.L., Finke C.M., Elala Y., Hanson C.A., Ketterling R.P., Gangat N., Pardanani A. (2016). Targeted deep sequencing in primary myelofibrosis. Blood Adv..

[79] Cruz-Molina S., Respuela P., Tebartz C., Kolovos P., Nikolic M., Fueyo R., Van Ijcken W.F.J., Grosveld F., Frommolt P., Bazzi H. (2017). PRC2 Facilitates the Regulatory Topology Required for Poised Enhancer Function during Pluripotent Stem Cell Differentiation. Cell Stem Cell.

[80] Margueron R., Reinberg D. (2011). The Polycomb complex PRC2 and its mark in life. Nature.

[81] Herrera-Merchan A., Arranz L., Ligos J.M., De Molina A., Dominguez O., Gonzalez S. (2012). Ectopic expression of the histone methyltransferase Ezh2 in haematopoietic stem cells causes myeloproliferative disease. Nat. Commun..

[82] Beguelin W., Popovic R., Teater M., Jiang Y., Bunting K.L., Rosen M., Shen H., Yang S.N., Wang L., Ezponda T. (2013). EZH2 is required for germinal center formation and somatic EZH2 mutations promote lymphoid transformation. Cancer Cell.

[83] Morin R.D., Johnson N.A., Severson T.M., Mungall A.J., An J., Goya R., Paul J.E., Boyle M., Woolcock B.W., Kuchenbauer F. (2010). Somatic mutations altering EZH2 (Tyr641) in follicular and diffuse large B-cell lymphomas of germinal-center origin. Nat. Genet..

[84] Guglielmelli P., Lasho T.L., Rotunno G., Mudireddy M., Mannarelli C., Nicolosi M., Pacilli A., Pardanani A., Rumi E., Rosti V. (2018). MIPSS70: Mutation-Enhanced International Prognostic Score System for Transplantation-Age Patients With Primary Myelofibrosis. J. Clin. Oncol..

[85] Bejar R., Stevenson K., Abdel-Wahab O., Galili N., Nilsson B., Garcia-Manero G., Kantarjian H., Raza A., Levine R.L., Neuberg D. (2011). Clinical effect of point mutations in myelodysplastic syndromes. N. Engl. J. Med..

[86] Gollner S., Oellerich T., Agrawal-Singh S., Schenk T., Klein H.U., Rohde C., Pabst C., Sauer T., Lerdrup M., Tavor S. (2017). Loss of the histone methyltransferase EZH2 induces resistance to multiple drugs in acute myeloid leukemia. Nat. Med..

[87] Guglielmelli P., Biamonte F., Score J., Hidalgo-Curtis C., Cervantes F., Maffioli M., Fanelli T., Ernst T., Winkelman N., Jones A.V. (2011). EZH2 mutational status predicts poor survival in myelofibrosis. Blood.

[88] Hirabayashi Y., Suzki N., Tsuboi M., Endo T.A., Toyoda T., Shinga J., Koseki H., Vidal M., Gotoh Y. (2009). Polycomb limits the neurogenic competence of neural precursor cells to promote astrogenic fate transition. Neuron.

[89] Neff T., Sinha A.U., Kluk M.J., Zhu N., Khattab M.H., Stein L., Xie H., Orkin S.H., Armstrong S.A. (2012). Polycomb repressive complex 2 is required for MLL-AF9 leukemia. Proc. Natl. Acad. Sci. USA.

[90] Su I.H., Basavaraj A., Krutchinsky A.N., Hobert O., Ullrich A., Chait B.T., Tarakhovsky A. (2003). Ezh2 controls B cell development through histone H3 methylation and Igh rearrangement. Nat. Immunol..

[91] Guglielmelli P., Zini R., Bogani C., Salati S., Pancrazzi A., Bianchi E., Mannelli F., Ferrari S., Le Bousse-Kerdiles M.C., Bosi A. (2007). Molecular profiling of CD34+ cells in idiopathic myelofibrosis identifies a set of disease-associated genes and reveals the clinical significance of Wilms’ tumor gene 1 (WT1). Stem Cells.

[92] Harada-Shirado K., Ikeda K., Ogawa K., Ohkawara H., Kimura H., Kai T., Noji H., Morishita S., Komatsu N., Takeishi Y. (2015). Dysregulation of the MIRLET7/HMGA2 axis with methylation of the CDKN2A promoter in myeloproliferative neoplasms. Br. J. Haematol..

[93] Boultwood J., Perry J., Pellagatti A., Fernandez-Mercado M., Fernandez-Santamaria C., Calasanz M.J., Larrayoz M.J., Garcia-Delgado M., Giagounidis A., Malcovati L. (2010). Frequent mutation of the polycomb-associated gene ASXL1 in the myelodysplastic syndromes and in acute myeloid leukemia. Leukemia.

[94] Boultwood J., Perry J., Zaman R., Fernandez-Santamaria C., Littlewood T., Kusec R., Pellagatti A., Wang L., Clark R.E., Wainscoat J.S. (2010). High-density single nucleotide polymorphism array analysis and ASXL1 gene mutation screening in chronic myeloid leukemia during disease progression. Leukemia.

[95] Carbuccia N., Murati A., Trouplin V., Brecqueville M., Adelaide J., Rey J., Vainchenker W., Bernard O.A., Chaffanet M., Vey N. (2009). Mutations of ASXL1 gene in myeloproliferative neoplasms. Leukemia.

[96] Carbuccia N., Trouplin V., Gelsi-Boyer V., Murati A., Rocquain J., Adelaide J., Olschwang S., Xerri L., Vey N., Chaffanet M. (2010). Mutual exclusion of ASXL1 and NPM1 mutations in a series of acute myeloid leukemias. Leukemia.

[97] Chen T.C., Hou H.A., Chou W.C., Tang J.L., Kuo Y.Y., Chen C.Y., Tseng M.H., Huang C.F., Lai Y.J., Chiang Y.C. (2014). Dynamics of ASXL1 mutation and other associated genetic alterations during disease progression in patients with primary myelodysplastic syndrome. Blood Cancer J..

[98] Gelsi-Boyer V., Trouplin V., Adelaide J., Bonansea J., Cervera N., Carbuccia N., Lagarde A., Prebet T., Nezri M., Sainty D. (2009). Mutations of polycomb-associated gene ASXL1 in myelodysplastic syndromes and chronic myelomonocytic leukaemia. Br. J. Haematol..

[99] Haferlach T., Nagata Y., Grossmann V., Okuno Y., Bacher U., Nagae G., Schnittger S., Sanada M., Kon A., Alpermann T. (2014). Landscape of genetic lesions in 944 patients with myelodysplastic syndromes. Leukemia.

[100] Huether R., Dong L., Chen X., Wu G., Parker M., Wei L., Ma J., Edmonson M.N., Hedlund E.K., Rusch M.C. (2014). The landscape of somatic mutations in epigenetic regulators across 1,000 paediatric cancer genomes. Nat. Commun..

[101] Makishima H., Jankowska A.M., McDevitt M.A., O’Keefe C., Dujardin S., Cazzolli H., Przychodzen B., Prince C., Nicoll J., Siddaiah H. (2011). CBL, CBLB, TET2, ASXL1, and IDH1/2 mutations and additional chromosomal aberrations constitute molecular events in chronic myelogenous leukemia. Blood.

[102] Sugimoto Y., Muramatsu H., Makishima H., Prince C., Jankowska A.M., Yoshida N., Xu Y., Nishio N., Hama A., Yagasaki H. (2010). Spectrum of molecular defects in juvenile myelomonocytic leukaemia includes ASXL1 mutations. Br. J. Haematol..

[103] Thol F., Friesen I., Damm F., Yun H., Weissinger E.M., Krauter J., Wagner K., Chaturvedi A., Sharma A., Wichmann M. (2011). Prognostic significance of ASXL1 mutations in patients with myelodysplastic syndromes. J. Clin. Oncol..

[104] Genovese G., Kahler A.K., Handsaker R.E., Lindberg J., Rose S.A., Bakhoum S.F., Chambert K., Mick E., Neale B.M., Fromer M. (2014). Clonal hematopoiesis and blood-cancer risk inferred from blood DNA sequence. N. Engl. J. Med..

[105] Jaiswal S., Fontanillas P., Flannick J., Manning A., Grauman P.V., Mar B.G., Lindsley R.C., Mermel C.H., Burtt N., Chavez A. (2014). Age-related clonal hematopoiesis associated with adverse outcomes. N. Engl. J. Med..

[106] Xie M., Lu C., Wang J., McLellan M.D., Johnson K.J., Wendl M.C., McMichael J.F., Schmidt H.K., Yellapantula V., Miller C.A. (2014). Age-related mutations associated with clonal hematopoietic expansion and malignancies. Nat. Med..

[107] Abdel-Wahab O., Adli M., LaFave L.M., Gao J., Hricik T., Shih A.H., Pandey S., Patel J.P., Chung Y.R., Koche R. (2012). ASXL1 mutations promote myeloid transformation through loss of PRC2-mediated gene repression. Cancer Cell.

[108] Nagase R., Inoue D., Pastore A., Fujino T., Hou H.A., Yamasaki N., Goyama S., Saika M., Kanai A., Sera Y. (2018). Expression of mutant Asxl1 perturbs hematopoiesis and promotes susceptibility to leukemic transformation. J. Exp. Med..

[109] Shi H., Yamamoto S., Sheng M., Bai J., Zhang P., Chen R., Chen S., Shi L., Abdel-Wahab O., Xu M. (2016). ASXL1 plays an important role in erythropoiesis. Sci. Rep..

[110] Yang H., Kurtenbach S., Guo Y., Lohse I., Durante M.A., Li J., Li Z., Al-Ali H., Li L., Chen Z. (2018). Gain of function of ASXL1 truncating protein in the pathogenesis of myeloid malignancies. Blood.

[111] Gelsi-Boyer V., Brecqueville M., Devillier R., Murati A., Mozziconacci M.J., Birnbaum D. (2012). Mutations in ASXL1 are associated with poor prognosis across the spectrum of malignant myeloid diseases. J. Hematol. Oncol..

[112] Wang J., Li Z., He Y., Pan F., Chen S., Rhodes S., Nguyen L., Yuan J., Jiang L., Yang X. (2014). Loss of Asxl1 leads to myelodysplastic syndrome-like disease in mice. Blood.

[113] Rinaldi L., Datta D., Serrat J., Morey L., Solanas G., Avgustinova A., Blanco E., Pons J.I., Matallanas D., Von Kriegsheim A. (2016). Dnmt3a and Dnmt3b Associate with Enhancers to Regulate Human Epidermal Stem Cell Homeostasis. Cell Stem Cell.

[114] Yang L., Rodriguez B., Mayle A., Park H.J., Lin X., Luo M., Jeong M., Curry C.V., Kim S.B., Ruau D. (2016). DNMT3A Loss Drives Enhancer Hypomethylation in FLT3-ITD-Associated Leukemias. Cancer Cell.

[115] Ley T.J., Ding L., Walter M.J., McLellan M.D., Lamprecht T., Larson D.E., Kandoth C., Payton J.E., Baty J., Welch J. (2010). DNMT3A mutations in acute myeloid leukemia. N. Engl. J. Med..

[116] Abdel-Wahab O., Pardanani A., Rampal R., Lasho T.L., Levine R.L., Tefferi A. (2011). DNMT3A mutational analysis in primary myelofibrosis, chronic myelomonocytic leukemia and advanced phases of myeloproliferative neoplasms. Leukemia.

[117] Stegelmann F., Bullinger L., Schlenk R.F., Paschka P., Griesshammer M., Blersch C., Kuhn S., Schauer S., Dohner H., Dohner K. (2011). DNMT3A mutations in myeloproliferative neoplasms. Leukemia.

[118] Russler-Germain D.A., Spencer D.H., Young M.A., Lamprecht T.L., Miller C.A., Fulton R., Meyer M.R., Erdmann-Gilmore P., Townsend R.R., Wilson R.K. (2014). The R882H DNMT3A mutation associated with AML dominantly inhibits wild-type DNMT3A by blocking its ability to form active tetramers. Cancer Cell.

[119] Yang L., Rau R., Goodell M.A. (2015). DNMT3A in haematological malignancies. Nat. Rev. Cancer.

[120] Challen G.A., Sun D., Jeong M., Luo M., Jelinek J., Berg J.S., Bock C., Vasanthakumar A., Gu H., Xi Y. (2012). Dnmt3a is essential for hematopoietic stem cell differentiation. Nat. Genet..

[121] Van den Akker E.B., Pitts S.J., Deelen J., Moed M.H., Potluri S., Van Rooij J., Suchiman H.E., Lakenberg N., De Dijcker W.J., Uitterlinden A.G. (2016). Uncompromised 10-year survival of oldest old carrying somatic mutations in DNMT3A and TET2. Blood.

[122] Young A.L., Challen G.A., Birmann B.M., Druley T.E. (2016). Clonal haematopoiesis harbouring AML-associated mutations is ubiquitous in healthy adults. Nat. Commun..

[123] Guryanova O.A., Shank K., Spitzer B., Luciani L., Koche R.P., Garrett-Bakelman F.E., Ganzel C., Durham B.H., Mohanty A., Hoermann G. (2016). DNMT3A mutations promote anthracycline resistance in acute myeloid leukemia via impaired nucleosome remodeling. Nat. Med..

[124] Yue L., Bartenstein M., Zhao W., Ho W.T., Han Y., Murdun C., Mailloux A.W., Zhang L., Wang X., Budhathoki A. (2017). Efficacy of ALK5 inhibition in myelofibrosis. JCI Insight.

[125] Chagraoui H., Komura E., Tulliez M., Giraudier S., Vainchenker W., Wendling F. (2002). Prominent role of TGF-beta 1 in thrombopoietin-induced myelofibrosis in mice. Blood.

[126] Evrard S., Bluteau O., Tulliez M., Rameau P., Gonin P., Zetterberg E., Palmblad J., Bonnefoy A., Villeval J.L., Vainchenker W. (2011). Thrombospondin-1 is not the major activator of TGF-beta1 in thrombopoietin-induced myelofibrosis. Blood.

[127] Chagraoui H., Tulliez M., Smayra T., Komura E., Giraudier S., Yun T., Lassau N., Vainchenker W., Wendling F. (2003). Stimulation of osteoprotegerin production is responsible for osteosclerosis in mice overexpressing TPO. Blood.

[128] Verstovsek S., Manshouri T., Pilling D., Bueso-Ramos C.E., Newberry K.J., Prijic S., Knez L., Bozinovic K., Harris D.M., Spaeth E.L. (2016). Role of neoplastic monocyte-derived fibrocytes in primary myelofibrosis. J. Exp. Med..

[129] Schneider R.K., Mullally A., Dugourd A., Peisker F., Hoogenboezem R., Van Strien P.M.H., Bindels E.M., Heckl D., Busche G., Fleck D. (2017). Gli1(+) Mesenchymal Stromal Cells Are a Key Driver of Bone Marrow Fibrosis and an Important Cellular Therapeutic Target. Cell Stem Cell.

[130] Van Bon L., Affandi A.J., Broen J., Christmann R.B., Marijnissen R.J., Stawski L., Farina G.A., Stifano G., Mathes A.L., Cossu M. (2014). Proteome-wide analysis and CXCL4 as a biomarker in systemic sclerosis. N. Engl. J. Med..

[131] Kramann R., Fleig S.V., Schneider R.K., Fabian S.L., DiRocco D.P., Maarouf O., Wongboonsin J., Ikeda Y., Heckl D., Chang S.L. (2015). Pharmacological GLI2 inhibition prevents myofibroblast cell-cycle progression and reduces kidney fibrosis. J. Clin. Investig..

[132] Moshai E.F., Wemeau-Stervinou L., Cigna N., Brayer S., Somme J.M., Crestani B., Mailleux A.A. (2014). Targeting the hedgehog-glioma-associated oncogene homolog pathway inhibits bleomycin-induced lung fibrosis in mice. Am. J. Respir. Cell Mol. Biol..

[133] Celik H., Koh W.K., Kramer A.C., Ostrander E.L., Mallaney C., Fisher D.A.C., Xiang J., Wilson W.C., Martens A., Kothari A. (2018). JARID2 Functions as a Tumor Suppressor in Myeloid Neoplasms by Repressing Self-Renewal in Hematopoietic Progenitor Cells. Cancer Cell.

[134] Fisher D.A.C., Miner C.A., Engle E.K., Hu H., Collins T.B., Zhou A., Allen M.J., Malkova O.N., Oh S.T. (2019). Cytokine production in myelofibrosis exhibits differential responsiveness to JAK-STAT, MAP kinase, and NFkappaB signaling. Leukemia.

[135] Martinez-Trillos A., Gaya A., Maffioli M., Arellano-Rodrigo E., Calvo X., Diaz-Beya M., Cervantes F. (2010). Efficacy and tolerability of hydroxyurea in the treatment of the hyperproliferative manifestations of myelofibrosis: Results in 40 patients. Ann. Hematol..

[136] Rampal R., Al-Shahrour F., Abdel-Wahab O., Patel J.P., Brunel J.P., Mermel C.H., Bass A.J., Pretz J., Ahn J., Hricik T. (2014). Integrated genomic analysis illustrates the central role of JAK-STAT pathway activation in myeloproliferative neoplasm pathogenesis. Blood.

[137] Harrison C., Kiladjian J.J., Al-Ali H.K., Gisslinger H., Waltzman R., Stalbovskaya V., McQuitty M., Hunter D.S., Levy R., Knoops L. (2012). JAK inhibition with ruxolitinib versus best available therapy for myelofibrosis. N. Engl. J. Med..

[138] Verstovsek S., Kantarjian H., Mesa R.A., Pardanani A.D., Cortes-Franco J., Thomas D.A., Estrov Z., Fridman J.S., Bradley E.C., Erickson-Viitanen S. (2010). Safety and efficacy of INCB018424, a JAK1 and JAK2 inhibitor, in myelofibrosis. N. Engl. J. Med..

[139] Austin R.J., Straube J., Bruedigam C., Pali G., Jacquelin S., Vu T., Green J., Grasel J., Lansink L., Cooper L. (2020). Distinct effects of ruxolitinib and interferon-alpha on murine JAK2V617F myeloproliferative neoplasm hematopoietic stem cell populations. Leukemia.

[140] Koppikar P., Bhagwat N., Kilpivaara O., Manshouri T., Adli M., Hricik T., Liu F., Saunders L.M., Mullally A., Abdel-Wahab O. (2012). Heterodimeric JAK-STAT activation as a mechanism of persistence to JAK2 inhibitor therapy. Nature.

[141] Doroshow D.B., Eder J.P., LoRusso P.M. (2017). BET inhibitors: A novel epigenetic approach. Ann. Oncol..

[142] Mascarenhas J., Kremyanskaya M., Hoffman R., Bose P., Talpaz M., Harrison C.N., Gupta V., Leber B., Sirhan S., Kabir S. (2019). MANIFEST, a Phase 2 Study of CPI-0610, a Bromodomain and Extraterminal Domain Inhibitor (BETi), As Monotherapy or “Add-on” to Ruxolitinib, in Patients with Refractory or Intolerant Advanced Myelofibrosis. Am. Soc. Hematol. Orlando.

[143] Harrison N., Patriarca A., Mascarenhas J., Kremyanskaya M., Hoffman R., Schiller G.J., Leber B., Devos T., Kabir S., Senderowicz A. (2019). Preliminary Report of MANIFEST, a Phase 2 Study of CPI-0610, a Bromodomain and Extraterminal Domain Inhibitor (BETi), in Combination with Ruxolitinib, in JAK Inhibitor (JAKi) Treatment Naïve Myelofibrosis Patients. Am. Soc. Hematol. Orlando.

[144] Lara-Astiaso D., Weiner A., Lorenzo-Vivas E., Zaretsky I., Jaitin D.A., David E., Keren-Shaul H., Mildner A., Winter D., Jung S. (2014). Immunogenetics. Chromatin state dynamics during blood formation. Science.

